# Applying STOPP Guidelines in Primary Care Through Electronic Medical Record Decision Support: Randomized Control Trial Highlighting the Importance of Data Quality

**DOI:** 10.2196/medinform.6226

**Published:** 2017-06-15

**Authors:** Morgan Price, Iryna Davies, Raymond Rusk, Mary Lesperance, Jens Weber

**Affiliations:** ^1^ LEAD Lab Department of Family Practice, Island Medical Program University of British Columbia Victoria, BC Canada; ^2^ University of Victoria Victoria, BC Canada

**Keywords:** electronic medical records, clinical decision support, randomized control trial, electronic prescribing, data quality

## Abstract

**Background:**

Potentially Inappropriate Prescriptions (PIPs) are a common cause of morbidity, particularly in the elderly.

**Objective:**

We sought to understand how the Screening Tool of Older People’s Prescriptions (STOPP) prescribing criteria, implemented in a routinely used primary care Electronic Medical Record (EMR), could impact PIP rates in community (non-academic) primary care practices.

**Methods:**

We conducted a mixed-method, pragmatic, cluster, randomized control trial in research naïve primary care practices. Phase 1: In the randomized controlled trial, 40 fully automated STOPP rules were implemented as EMR alerts during a 16-week intervention period. The control group did not receive the 40 STOPP rules (but received other alerts). Participants were recruited through the OSCAR EMR user group mailing list and in person at user group meetings. Results were assessed by querying EMR data PIPs. EMR data quality probes were included. Phase 2: physicians were invited to participate in 1-hour semi-structured interviews to discuss the results.

**Results:**

In the EMR, 40 STOPP rules were successfully implemented. Phase 1: A total of 28 physicians from 8 practices were recruited (16 in intervention and 12 in control groups). The calculated PIP rate was 2.6% (138/5308) (control) and 4.11% (768/18,668) (intervention) at baseline. No change in PIPs was observed through the intervention (P=.80). Data quality probes generally showed low use of problem list and medication list. Phase 2: A total of 5 physicians participated. All the participants felt that they were aware of the alerts but commented on workflow and presentation challenges.

**Conclusions:**

The calculated PIP rate was markedly less than the expected rate found in literature (2.6% and 4.0% vs 20% in literature). Data quality probes highlighted issues related to completeness of data in areas of the EMR used for PIP reporting and by the decision support such as problem and medication lists. Users also highlighted areas for better integration of STOPP guidelines with prescribing workflows. Many of the STOPP criteria can be implemented in EMRs using simple logic. However, data quality in EMRs continues to be a challenge and was a limiting step in the effectiveness of the decision support in this study. This is important as decision makers continue to fund implementation and adoption of EMRs with the expectation of the use of advanced tools (such as decision support) without ongoing review of data quality and improvement.

**Trial Registration:**

Clinicaltrials.gov NCT02130895; https://clinicaltrials.gov/ct2/show/NCT02130895 (Archived by WebCite at http://www.webcitation.org/6qyFigSYT)

## Introduction

### Potentially Inappropriate Prescriptions

Adverse Drug Events (ADEs), injuries, and deaths resulting from the administration of a medication [1-3], are a leading cause of iatrogenic morbidity and mortality. Canadian adverse event rates are estimated at 185,000 annually, with 70,000 being potentially preventable [4,5]. The ADE rates are similar in the United States, with the Institute of Medicine estimating that 100,000 preventable deaths occur per year in the United States [6,7]. Medication errors have a greater impact on vulnerable populations such as the elderly, who have significant illness burdens and are often taking a number of medications [8]. It has been reported that 27% of the elderly are on 5 or more medications [8]. The cost of ADEs in seniors is high: over Can $35 million annually in Canada [9]. Avoiding inappropriate prescriptions is one important approach to avoiding predictable ADEs among older people [10]. Effective prevention should involve primary care.

### Criteria for the Screening Tool of Older People’s Prescriptions

Several groups have attempted to reduce inappropriate prescriptions for the elderly, creating a number of guidelines and criteria to help prescribers use a rational approach to drug prescriptions for the elderly [11]. The Beers criteria [12-16] were developed to support clinicians, and more recently, the STOPP criteria (screening tool of older people's prescriptions) have been developed [17,18]. The STOPP criteria [17-19] consist of 65 recommendations (114 in version 2 [19]) that support evidence-based, individualized prescribing practices among patients 65 and over. The criteria take into account a range of salient patient features to predict potentially inappropriate prescriptions. A systematic review showed that STOPP version 1 was more sensitive than Beers in identifying inappropriate prescribing [11]. The majority of STOPP literature is focused on long-term care and hospital settings [12,14-16,20]. Less work has been done with STOPP in primary care [21,22], even though preventable ADEs are common and serious in this setting [4,23]. In response, this study set out to measure the impact of using the STOPP criteria in primary care, where the majority of prescriptions occur.

### Promise of Decision Support

Clinical Decision Support (CDS) aids clinicians and patients in making appropriate decisions in care. In primary care, CDS is often embedded into Electronic Medical Records (EMRs). There is promise for CDS in improving quality of care in general and prescribing in particular, with 66% of studies on prescribing systems showing positive outcomes [24]. Although there is promise in these tools, the benefits of using these tools are not consistently realized [25,26]. Studies, such as the MOXXI study, have shown variable responses to CDS for prescribing [27]. In some cases, the user experience of CDS tools is poor enough that alerts are overridden [1,3], the use of CDS and electronic tools have facilitated errors [4], or the CDS tools have had unintended consequences [28,29]. There is a pressing need to improve decision support tools for providers in order to better realize the expected benefits and reduce serious, unintended consequences.

Achieving impact with CDS tools is not without challenges. There are many “grand challenges” for CDS [30] such as development of content, making content available through Web-based systems, and user experience. The American Medical Informatics Association’s position paper on CDS design recommends CDS tools that better summarize and prioritize recommendations to reduce the cognitive burden on clinicians and maintain efficiency [31]. SAFER guidelines have been developed to “empower organizations to work with internal or external stakeholders on optimizing [EMR] functionality” [32]. Data quality is often discussed in terms of data use in research [33]; however, data quality is also foundational to CDS [34].

### Research Objective

Through this mixed-method study, we sought to answer the following overarching question:

How can an existing clinical decision support tool implement a complex set of evidence-based rules into primary care clinical practice and how does this impact prescribing?

We considered this question in the context of a primary care EMR with CDS. The EMR was able to be populated by a Web-based decision support application to provide rules. We sought to understand the answers to the question through a combination of EMR data quality probes and participant interviews.

## Methods

This was a mixed-method study divided into two phases: (1) A randomized control trial and (2) A qualitative reflection by participants on the results.

### Phase 1: Pragmatic Randomized Control Trial

Phase 1 was a prospective, intention-to-treat, un-blinded, cluster randomized trial. The primary outcome measure was the *change* in rate of STOPP-defined PIPs as documented in the OSCAR EMR in the intervention group as compared with the control group.

#### Participants

##### Inclusion Criteria

The participants were primary care physicians in British Columbia providing office-based care to patients 65 and over, using the open source OSCAR EMR developed by McMaster University and the OSCAR community (version 12.x) for at least 12 months (this was to provide enough time for medications to be consistently documented in the EMR), and who were part of or willing to be part of the University of British Columbia’s Department of Family Practice Research Network.

##### Exclusion Criteria

Providers who do not provide longitudinal care (eg, walk in clinics) or only hospital care, who do not use OSCAR for writing prescriptions, or who provide care to a younger population (eg, a maternity clinic) were excluded from the study.

##### Sample Size

Sample size was calculated assuming a PIP rate of 20% [10,27,35] and an expected relative reduction of 20% in PIPs (absolute reduction of 4%). Using a power of 0.8 and alpha=0.05 and estimating that two practices may be lost to follow up, we predicted the need for 12 practices in each arm and 900 encounters per arm with patients 65 years and over.

#### Recruitment and Randomization

Providers were recruited through the OSCAR Canada User Society’s mailing list and the 2014 OSCAR EMR national user meeting (over 100 people were made aware of the study). Potential participants were screened by the primary investigator on the phone or in person to ensure they met the criteria.

Clinics were randomly assigned (equal distribution by clinic) to the intervention or control groups using a random number generator that generated the list prior to the recruitment and allocation of participants. Randomization was stratified into small (<4 physicians) and large clinics (≥4 physicians).

All physicians (in the control and intervention groups) received the same orientation to the purpose of the study, the nature of the STOPP criteria, and what was being measured. The intervention group also received assistance in activating the STOPP criteria in their CDS tools. The control group was invited to have the STOPP guidelines activated after the study and the guidelines were made freely available to the OSCAR community after the study.

#### Intervention

**Figure 1:**
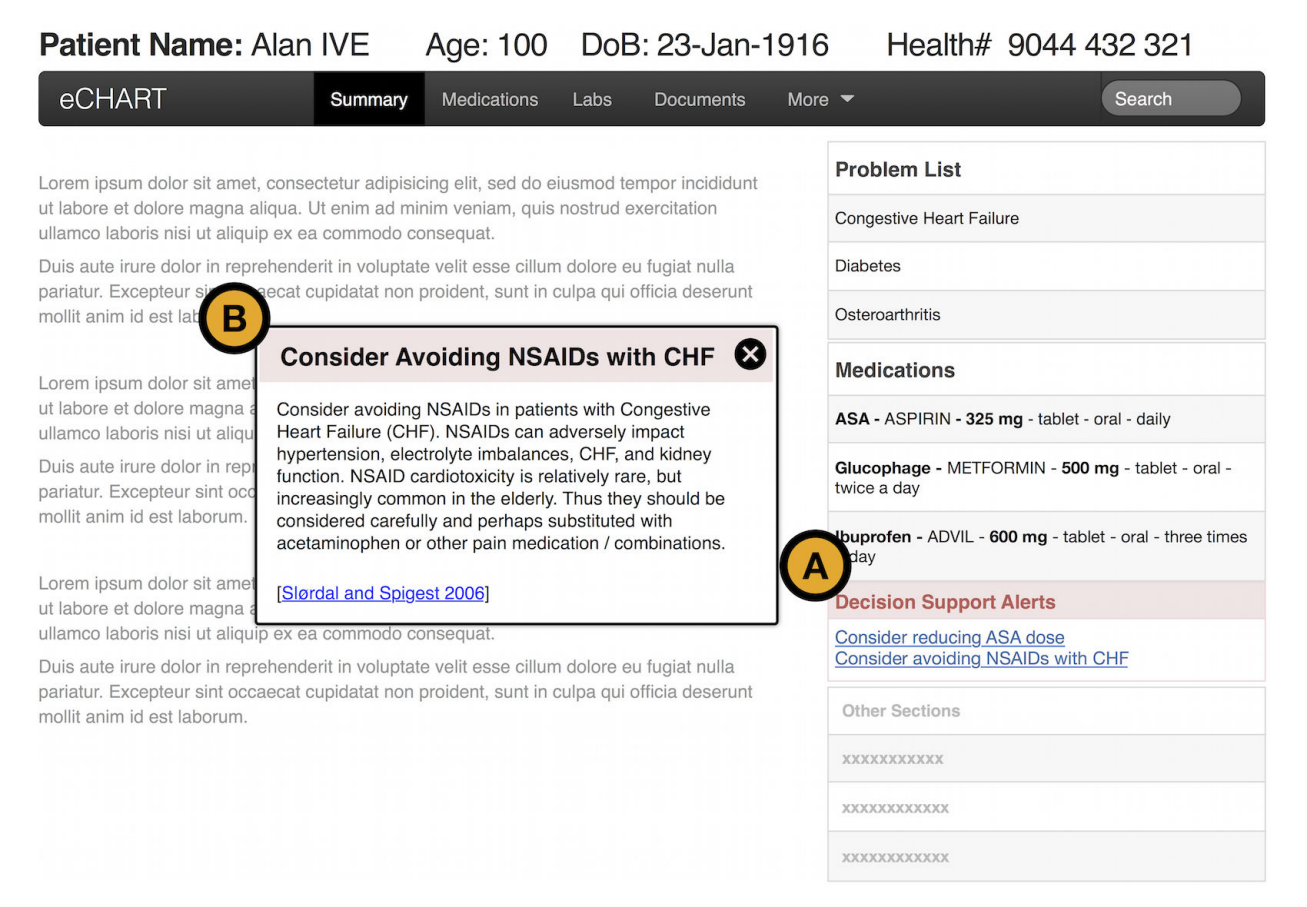
Wireframe of the CDS alerts in the EMR. (A) on the right is the panel that lists patient specific alerts. From that panel, users can click a title and get (B), the detail of the alert that pops up when clicked.

The intervention group received the STOPP guidelines content in their EMR, whereas the control group did not. The STOPP guidelines leveraged the existing CDS engine of the EMR. These additional guidelines provided suggestions to the providers when specific criteria were met for an individual patient being seen. The EMR showed patient specific guideline recommendation titles in a text window in the side bar of the patient’s chart. These titles could be clicked on for more information (Figure 1).

Implementation at the user level was required in the intervention group and facilitated by one of the authors (ID). Participants were instructed and walked through how to turn on the STOPP rules by trusting the STOPP content in the Clinical Decision Support (CDS) module. This downloaded and activated the STOPP rules for that user for the duration of the trial.

A subset of 40 STOPP rules (that were found in both STOPP v1 and v2) was developed for the EMR CDS rules engine. The rules and the network queries (which measured the outcomes as PIP rates) were generated from the same logic files to ensure consistency between the EMR CDS rules and the network measurements.

The 40 STOPP rules were successfully modeled and implemented for this study. It was not possible to create all STOPP rules due to features in the EMR or network query engine. STOPP rules that were not included contained concepts such as duration of combined prescriptions or dose thresholds that could not be modeled in one of the two components (eg, the EMR guideline logic or the network query). Shortly before the study was to start, version 2 of the STOPP rules were published. Rules from version 1 that were removed in version 2 were removed from our study.

#### Primary Outcome Measure

The primary outcome measure was the difference in change in measured PIP rates between the intervention and control groups before the intervention as compared with the difference after the intervention period.

#### Data Quality Probes

To provide context and estimate the validity of answers, a set of 13 data quality probes (DQ probes) were created. These assessed the data quality of demographics, medications, and the problem list—the three areas that were in the control of the clinic and related to the STOPP criteria. All DQ probes considered only those patients that had had an encounter at the clinic in the last two years. The DQ probes list is shown in Table 2.

### Data Collection

#### Phase 1: Collection of Physician Information

All the participants completed a survey describing their practice at the start of the study.

#### Measurement of Potentially Inappropriate Prescriptions

Measurement of PIPs and DQ probes was completed using the UBC Department of Family Practice research network, which was developed with the Physicians Data Collaborative of British Columbia. The network is based on hQuery, an open source tool that is freely available on GitHub. The research network is designed to distribute querying of EMR health data without collection and storage of patient level data. Only aggregate data (ie, summary answers to queries) are collected in the central Hub [36]. Patient privacy was maintained through the network as only practice level aggregate answers were returned through the network.

Baseline Rates of Potentially Inappropriate Prescriptions

Queries were run at the start of the study for each clinic to provide baseline data of the 16 weeks preceding the study.

Intervention Rates of Potentially Inappropriate Prescriptions

Queries were repeated after the 16-week intervention period to assess PIP rates for the intervention period.

### Data Analysis

PIPs for each of the STOPP rules and DQ probes were measured for each clinic. Statistical regression models (which account for the clustered nature of the data) were fit to assess the primary outcome (geeglm in R package geepack) [37].

All clinics received an individualized summary of the findings from the study including PIP rates, most common PIPs in their practice, and highlights of data quality in their EMRs.

#### Phase 2: Explaining Findings and Understanding Experiences

Phase 2 consisted of one-hour semistructured interviews with physicians in the intervention group. These reflection sessions encouraged physicians to explain the findings. The findings for the study were shared, including physicians’ own clinic specific summaries, and they were asked to reflect on the results and describe their experiences. Participants were invited to provide information on how to improve the tools in the EMR.

This study was registered at clinical trials.gov (NCT02130895) and received clinical ethics approval from the University of British Columbia (H14-00797).

## Results

### Participation

**Figure 2:**
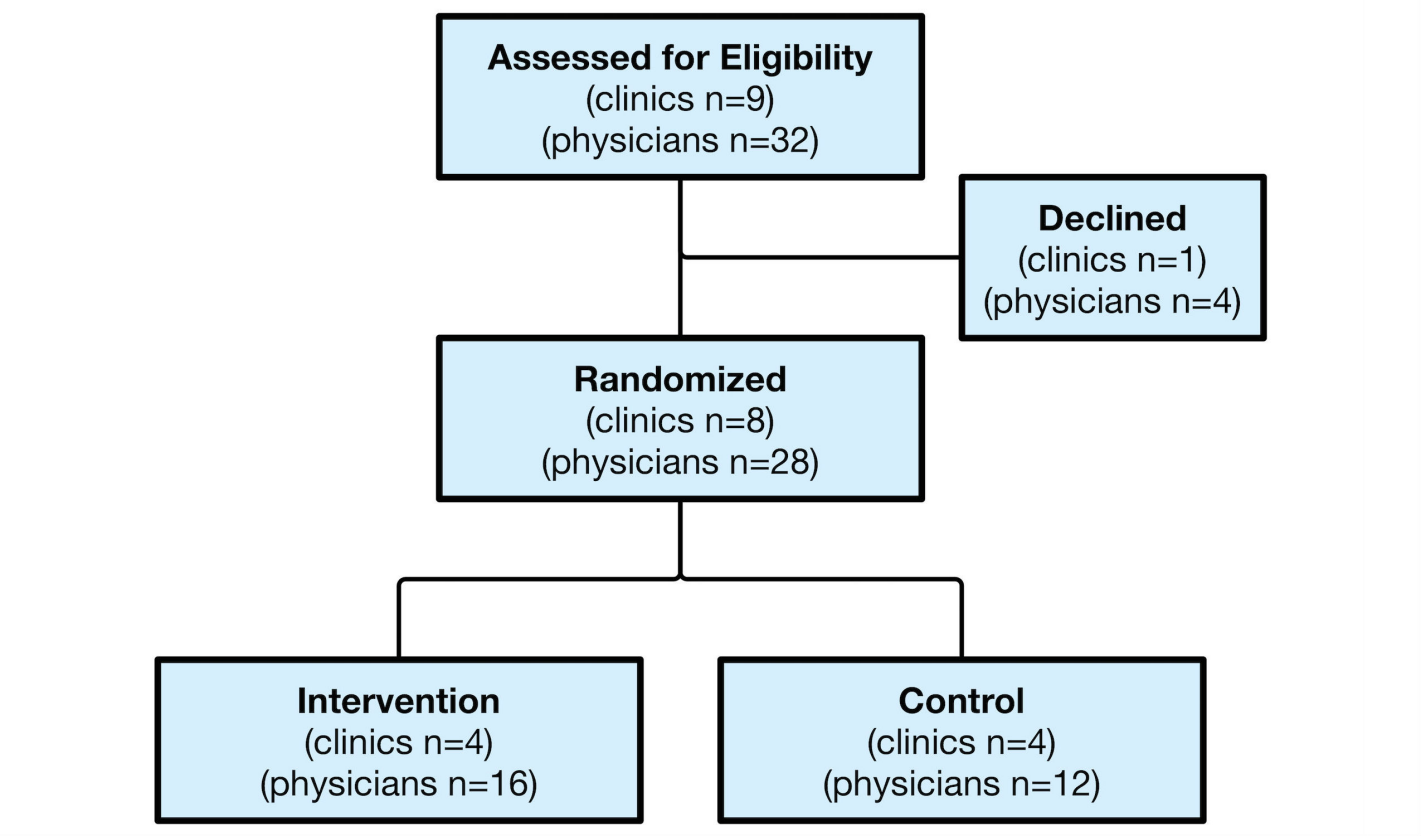
CONSORT figure. 28 physicians in 8 clinics were recruited into the study. 1 clinic declined to participate during recruitment. No clinics or physicians were lost to follow up during the trial.

The study was completed from February-October, 2015. A total of 8 clinics were engaged in the study (9 were approached, but one declined because of technical reasons with their EMR) and randomization occurred at the clinic level. Twenty-eight physicians across the eight practices consented in person. None were lost to follow up (Figure 2). One participant reported a technical problem with the EMR that was thought to be related to the intervention and later discovered to be unrelated.

### Phase 1 Results

The control group saw 1086 patients who could have triggered a PIP during the baseline period and 1204 during the treatment period. In the control group, there were 138 PIPs (out of a possible 5308 that could have been triggered) during baseline and 157 PIPs (out of a possible 5792) during treatment.

The intervention group saw 3556 patients who could have triggered a PIP during the baseline period and 3621 during the treatment period. In the intervention group, there were 742 PIPs during baseline (out of a possible 18,331) and 768 PIPs during treatment (out of a possible 18,668). There was an initial difference between the two groups (2.6% and 4.0% of prescriptions were flagged as PIPs in the control and intervention groups, respectively).

**Table 1:** Rates of PIPs in the control and intervention groups before and during the treatment periods.

Rates	Intervention, %	Control, %
Before treatment (baseline)	4.0	2.6
During treatment	4.1	2.7
Change in potentially inappropriate prescriptions (PIP)rates	0.1	0.1

Both groups saw similar numbers of patients during the study (44,290 in the control group and 37,615 in the intervention group); however, the intervention group had a greater proportion of patients who were 65 years and older (control 5% vs intervention 19%).

The regression analysis of PIPs showed no significant difference in change of recorded PIPs in the control group versus the intervention group (*P*=.80).

**Table 2:** Data quality probes assessing demographics, medications, and problem list usage.

Data quality probe description	Intervention, %	Control, %
What percentage of patients, flagged as active, had at least one encounter in the past 24 months?	35.7	28.6
What is the percentage of patients, calculated as active, with no documented gender?	0.3	0.5
What percentage of patients, calculated as active, has an invalid date of birth?	0.0	0.0
What percentage of patients, calculated as active, has no documented date of birth?	0.0	0.0
What percentage of current medications is coded?	82.3	79.0
What percentage of patients, calculated as active, has no current medications?	69.9	84.6
What percentage of problems on the problem list, documented in the past 12 months, has a diagnostic code?	100.0	100.0
What percentage of patients, aged 12 years and over and calculated as active, has at least one documented problem on the problem list (documented in the past 12 months)?	12.3	3.5
What percentage of patients, calculated as active and aged 12 years and over, has Diabetes on the problem list?	5.3	3.5
Of patients currently on Tiotropium medication, what percentage has “COPD”^a^ on the problem list?	48.1	72.7
Of patients currently on Levothyroxine medication, what percentage has “Hypothyroidism” on the problem list?	22.7	24.3
Of patients currently on anti-gout medication, what percentage has “Gout” on the problem list?	8.4	12.2

^a^COPD: chronic obstructive pulmonary disease.

### Data Quality Probes

EMR data quality was estimated using a set of DQ probes that were executed in the same way as PIP rates were evaluated. The DQ probes were used to assess completeness, correctness, and concordance.

Both groups had a large number of active patients (an average of 68.2%) that were documented as active according to the EMR but that had not had an appointment in at least two years. (Note: STOPP queries were designed only to look at patients who had had an encounter during either the baseline or intervention period). We found that 79-82% of prescriptions were coded, with the remainder being free text; however, there were a high number of patients who were not on any active medications in both groups. All documented problems on the problem list were coded (this was a requirement for the working of the EMR); however, only a small number of patients (12.3% in the intervention group and 3.5% in the control group) had at least one problem on the problem list. In the control group, interestingly, nearly all the patients were also diabetic. The three DQ probes that relate diagnosis to medication use (COPD, hypothyroidism, and gout) all showed that the coded problem list was under-utilized.

The software engineers on this project confirmed the results of the study’s queries by using an alternate query method to ensure that the query logic was running correctly. These queries confirmed the above findings.

### Phase 2 Findings

Total of 5 physicians across 3 of the 4 intervention group clinics participated in phase 2. Two of the participants had discussed the study and the phase 2 interview with their colleagues in preparation for the meeting and shared their collective thoughts. Three themes emerged from the interviews.

#### Alert Awareness

All phase 2 participants felt that they were aware of the STOPP alerts (participants were not blinded to being in the intervention group). However, although they all felt that they had seen “some” CDS alerts, they felt they might not have *consistently* seen them.

#### Workflow and Display

The location on screen and the workflow were thought to be barriers. The STOPP criteria, as they had more complex rules, were implemented differently and displayed in a separate location to simple drug alerts. This often meant that the user would need to tab between screens and refresh screens. Participants preferred a single location for all medication related alerts, regardless of the logic behind those alerts.

#### Study Disruptiveness

Finally, the participants reflected on the disruption caused by the study to their practice. All of them agreed that the disruption caused by this kind of study was minimal and that they would all participate in future studies.

## Discussion

We sought to obtain an answer to the following question:

How can an existing clinical decision support tool implement a complex set of evidence-based rules into primary care clinical practice and how does this impact prescribing?

A total of 40 STOPP criteria were implemented in the primary care EMR. There were some limitations in the CDS module logic that prevented some rules from being implemented, such as being able to calculate the duration for which a patient had been on a medication over multiple prescriptions. However, the randomized trial component of this study was unable to show a significant change in PIPs rates that could be attributed to the STOPP guidelines as implemented. There are at least two reasons for this.

First, the rate of measured PIPs was lower than expected when compared with PIP rates found in the literature [10,27,35]. With measured PIP rates of 2.6% and 4.0%, it was not possible to see the decrease in PIPs expected from other studies. The DQ probes begin to shed light on why the measured PIP rate is lower than expected. The areas of the EMR that were used by the STOPP rules (eg, the coded problems on the problem list and coded prescriptions that were up to date) were utilized with less frequency than expected and, thus, the CDS rules were not fired as often. For example, we see that 92% of patients had no diagnosis input into their coded problem list, which is a rate of use that is lower than anticipated. Gaps in EMR data quality are as important today as they were twenty years ago [38]. While electronic medical record data are increasingly available and easy to access, the data quality is increasingly the challenge [39]. This is a key point highlighted by this study that should be considered by implementers and decision makers. Even though this study was completed with people using an established EMR that has been widely adopted in Canada and had been adopted in the participating clinics for some time, the data was not fit for use in the CDS.

Data quality is often talked about in terms of data warehouses [33], connecting multiple systems [40], and big data [41]. This study also highlights that data quality is a limiter when applying computational interventions that are designed to support improvements in care for individual patients. Missing, uncoded, or variably documented (eg, in another location within the EMR) data may well have been the limiter in this study.

Second, the participants in phase 2 discussed some challenges with the CDS. For example, the workflow of the STOPP alerts was less than ideal: for technical reasons, the STOPP rules were presented in the main chart and not in the prescription module. The guideline tool did not have a clear way to support users in prioritizing suggestions and alerts as recommended [42]. The alerts were positioned near the bottom of the screen and the users felt that, while they were aware of them, they were small, in a list with other reminders, and could potentially be missed.

Despite the negative result, there are still several valuable lessons that can be learned from this pragmatic trial.

### Lessons Learned

#### Translating Rules for the Screening Tool of Older People’s Prescriptions into Electronic Medical Record Algorithms

In general, the STOPP rules were well specified and computable definitions that could be created for most rules. However, in many cases, definitions had to be refined based on the logic features and data accessible in the EMR. The STOPP rules were not defined using specific medical terminologies (eg, International Classification of Disease (ICD) codes) and these had to be developed for this study. This gap is not unique to STOPP rules; indeed, others have had similar challenges in translating clinical guidelines into computer interpretable rules or alerts [43]. However, we recommend that future versions of STOPP and other recommendations consider using terminologies in their definitions to aid in consistent translation into computable forms. Finally, the CDS engine was not able to access all EMR data elements described in the STOPP criteria and several rules were excluded.

#### Data Quality

This was a pragmatic trial. We wanted to see the impact of implementation of CDS in nonacademic practices that had not gone through extensive data quality improvement training. This would better predict impact of CDS tools in real world settings. We discovered that 77% of patients were not on medications that could be queried. Approximately 20% of prescriptions were documented in the prescription writer without codes that could be queried, indicating custom medications (ie, free text) that would further limit the ability of the CDS. Over 92% of patients had no coded problems on the problem list (these were queries of the practice, not just the study population). As these were two data sources for many of the queries, data quality was one of the limiting factors to the triggering of the CDS guidelines. This would likely impact other similar uses of CDS without remediation. The EMR allowed for free text in several places (eg, within encounter notes) and so it would be possible for many more items to be recorded in the EMR than could be queried in a coded manner, if the respective EMR components were not used as intended.

Participants in phase 2 were generally unaware of these data quality issues. Previous work has highlighted that physicians often think of the EMR as an “electronic paper record” [44] and do not consider the downstream impacts of not using or inappropriately using components of the EMR. There is an ongoing need for more general education around the use of EMR and EMR data for primary care physicians.

#### EMR Workflow Limitations

In this study, the EMR could not integrate complex CDS rules into the prescribing module. Thus, STOPP alerts appeared in the main screen of the patient chart. Future implementations should allow for better integration into key workflows, such as prescribing. Furthermore, while the guideline engine did allow for summary and additional information to be displayed on user request, it did not allow for easy actions to be performed (eg, discontinuing a medication that triggered the STOPP guideline). There was an EMR workflow that users could use that would skip the screen where the CDS STOPP rules were displayed; however, participants in phase 2 stated that this was not a typical workflow. This issue has been addressed in an upcoming version of the EMR.

#### Study Implementation

One of the guiding principles when developing this study was to implement and run the study with as little disruptive impact on practicing physicians as possible. Feedback from the participants was positive in terms of ease of study participation. However, an important matter to explore in future work is the minimum amount of training needed to achieve sufficient improvements in data quality to achieve benefits from the application of complex rules like STOPP. This has implications not just for future studies but also for the implementation of future CDS as part of EMR requirements and quality improvement initiatives.

#### EMRs Permit Variable Workflows

Although we confirmed subjectively through phase 2 interviews that practitioners saw the guidelines and had the opportunity to act, we did not have a mechanism to proactively measure individual workflows in the EMR. It is possible (eg, through quick links for direct medication renewals) to avoid the triggering of the EMR guideline module where the STOPP rules reside. We were not able to measure how often this quick link or other paths might have been used. This information would be helpful in understanding how to redesign the EMR and other clinical information systems in the future.

#### Embedding Knowledge Translation

This study was designed with two knowledge translation (KT) partners: the EMR and the research network. The STOPP alerts and network queries were developed with these two groups and the materials were provided to each group freely at the end of the study. This proved to be an effective way of engaging with participants and partners and ensuring that the knowledge and artifacts from the study have future application. It was an excellent model to engage both the partners and the end-users in the study as the providers understood that their participation would allow for greater and ongoing impacts in the community.

### Study Limitations

The study had several limitations, which were as follows. (1) Participants were not blinded to which arm of the study they were in. (2) Physicians and clinics volunteered to participate in the network and the study. (3) Because of the timing of the study, the newest version of the particular EMR was not yet installed for the study clinics. This resulted in using an outdated user interface model. The newer EMR has addressed several workflow issues related to CDS and prescribing. (4) Only 40 STOPP study criteria were implemented. (5) Data quality probes were of a more general nature and not specific to the age range of this study’s patient population.

### Future Direction

Data quality is a key issue for the use of CDS tools, especially outside of large academic centers where there may be additional resources to improve data quality. The authors have begun to consider data quality by design as an engineering framework that can be applied in the real world [45]. Given the highlights related to workflow and CDS from this study, current work is exploring new user interface designs that support different paradigms for CDS [46] in prescribing. These design ideas are being shared with EMR vendors. As newer versions of the EMR and other study components are engineered and adopted, repeating the study will allow for some level of comparison to assess the changes in design.

### Conclusions

This pragmatic study intentionally implemented a subset of STOPP prescribing guidelines into nonacademic primary care offices with minimal training and disruption. One of the limitations discovered was the data quality in the EMR databases. The rates of measured potentially inappropriate prescriptions was limited and, thus, the rate at which the decision support would be triggered was limited by insufficient use of the EMR components that were connected to the decision support system. Further, this study provides more evidence to support the need to carefully design the workflows of the EMR tools that will support quality. As decision makers create policy to implement tools in EMRs such as decision support, careful attention will be required to ensure that practices and their data are ready to adopt these tools.

## Abbreviations

CDS: clinical decision support
CDSS: clinical decision support system
COPD: Chronic Obstructive Pulmonary Disease
DQ: data quality
ICD: International Classification of Disease
EMR: electronic medical record
PIP: potentially inappropriate prescriptions
RCT: randomized controlled trial
STOPP: screening tool of older people’s prescriptions

## Appendix

Multimedia Appendix 1 CONSORT EHealth Checklist (V 1.6.1).
